# Perioperative factors that are significantly correlated with final visual acuity in eyes after successful rhegmatogenous retinal detachment surgery

**DOI:** 10.1371/journal.pone.0184783

**Published:** 2017-09-13

**Authors:** Misato Kobayashi, Takeshi Iwase, Kentaro Yamamoto, Eimei Ra, Kenta Murotani, Hiroko Terasaki

**Affiliations:** 1 Department of Ophthalmology, Nagoya University Graduate School of Medicine, Nagoya, Japan; 2 Center for Advanced Medicine and Clinical Research, Nagoya University Hospital, Nagoya, Japan; Massachusetts Eye & Ear Infirmary, Harvard Medical School, UNITED STATES

## Abstract

**Purpose:**

To determine the perioperative factors that are significantly correlated with the final visual acuity following reattachment of a macula-off rhegmatogenous retinal detachment (RRD) by vitrectomy.

**Methods:**

Twenty-nine eyes of 29 patients with a successfully reattached RRD by vitrectomy were retrospectively analyzed. Spectral-domain optical coherence tomographic images of the macular regions were used to measure the thicknesses of the retinal layers and the integrity of the microstructures of the photoreceptors at 2 weeks, 1, 2, 3, 6, 9, and 12 months following the vitrectomy. The best-corrected visual acuities (BCVA) were evaluated at the same times.

**Results:**

The improvement of the BCVA from the preoperative BCVA to that at postoperative Week 2 (-0.67 ± 0.69 logMAR units) was the largest change between adjacent observation periods for the entire study duration. It was significantly greater than the improvement between Week 2 and Month 12 (-0.32 ± 0.22 logMAR units; *P*<0.001). The thickness of the ellipsoid zone (EZ)-retinal pigment epithelium (RPE) increased significantly with time (*P*<0.001). The final BCVA was significantly correlated with the BCVA at Week 2 (r = 0.61, *P*<0.001), the EZ-RPE thickness at Week 2 (r = -0.40, *P* = 0.035), the integrity of the external limiting membrane (ELM) (r = -0.61, *P* = 0.003), and an intact EZ (r = -0.66, *P* = 0.001) at Week 2. Multiple stepwise regression analyses of the final BCVA showed that the BCVA at Week 2 (*P* = 0.017) and the integrity of the EZ at Week 2 (*P* = 0.006) were independent predictors of the final BCVA.

**Conclusions:**

The significantly better BCVA and presence of an intact EZ at 2 weeks following vitrectomy and their significant correlations with the BCVA at Month 12 indicate that these perioperative values can be used to predict the BCVA at Month 12 after a reattachment of macula-off RRD following vitrectomy.

## Introduction

A rhegmatogenous retinal detachment (RRD) is a sight-threatening retinal disease, and currently, the only treatment for a RRD is a surgical reattachment of the retina. [1] Despite the high level of anatomic success of retinal reattachment surgery, [2–4] the improvement of the central vision may remain compromised because of functional damages to the retina during the detachment period. [5, 6]

The preoperative factors reported to be associated with the functional recovery following reattachment of a macula-off RRD include the preoperative best-corrected visual acuity (BCVA), [7] duration of the macular detachment, [8] [9] height of the macular detachment, [10] [11] and age of the patient. [4] However, these preoperative factors can depress the preoperative vision in eyes with macula-off RRD. Thus, clinicians cannot predict the postoperative visual outcomes accurately in eyes with macula-off RRD. A search of Medline with key words, ‘macula-off RRD’, ‘BCVA’ and ‘vitrectomy’, extracted many publications which examined the recovery of the BCVA during the postoperative period. However, none of the studies examined the BCVA at the very early postoperative period, e.g., at 2 weeks. Thus, it is still not known what perioperative factor is the best predictor of the final BCVA after successful reattachment of eyes with a macula-off RRD.

Currently, optical coherence tomography (OCT) is being used to evaluate the microstructures of the photoreceptors. Many OCT studies have demonstrated that the integrities of the retinal hyperreflective outer bands, e.g., the external limiting membrane (ELM), the ellipsoid zone (EZ), and cone interdigitation zone (CIZ), are significantly correlated with the BCVA following a retinal reattachment.[12–18] However, it is difficult to evaluate these retinal outer bands in the preoperative or the very early postoperative period because of the distortions of the detached retina.

Recent improvements in the resolution of spectral-domain (SD)-OCT instruments have made it possible to obtain clearer images of the foveal microstructures. The findings have allowed clinicians to follow the recovery process after reattachment surgeries more precisely and accurately. It has been reported that there was a significant correlation between the presence of a foveal bulge and good BCVA after successful RRD reattachment by vitrectomy. [18, 19] However, a foveal bulge is first observed usually postoperatively at 2 month in eyes with successful macula-off RRD surgery. [18, 19] This indicates that the presence of a foveal bulge is probably not the best structure to use at the very early postoperative times to predict the final BCVA.

Dell’Omo et al.[20] and Terauchi et al.[21] performed serial evaluations of changes in the retinal layer thicknesses at the same location by SD-OCT, and they reported a significant increase in the thickness of several central retinal layers one month after the reattachment surgery. The distance between the EZ and RPE, the EZ-RPE thickness, was reported to be significantly correlated with the BCVA at 1 month following successful reattachment of a macula-off RRD. [20] However, there have been no reports evaluating the retinal layer thickness and BCVA in the very early postoperative stage, e.g., less than 1 month following the surgery.

Thus, the purpose of this study was to evaluate the BCVA, the integrity of the retinal outer microstructures, and retinal layer thicknesses beginning 2 weeks after a successful reattachment by vitrectomy. In addition, we also determined the factors in the perioperative period that were significantly correlated with a good BCVA at the final examination after a reattachment of a macula-off detached retina by vitrectomy.

## Patients and methods

### Ethics statement

The procedures used in this observational, comparative, single-center study conformed to the tenets of the Declaration of Helsinki, and they were approved by the Institutional Review Board and Ethics Committee of the Nagoya University Graduate School of Medicine. Written informed consent was obtained from all patients.

### Subjects

We reviewed the medical records of all patients who had undergone successful RRD repair by vitrectomy at the Nagoya University Hospital from January 2012 to January 2015. All patients had a retinal detachment involving the macula, i.e., a macula-off RRD, and they were evaluated by SD-OCT macular scans pre- and postoperatively.

All patients had a comprehensive ophthalmic examination including measurements of the BCVA, intraocular pressure (IOP), and axial length. They also had slit-lamp, ophthalmoscopic, and SD-OCT examinations before and at 2 weeks, and 1, 2, 3, 6, 9, and 12 months after the surgery. The Snellen VA values were converted to the logarithm of the minimum angle of resolution (logMAR) units for the statistical analyses.

### Surgical techniques

Standard 3-port pars plana vitrectomy was performed with 25-gauge instruments as described in detail previously. [18] None of the patients had concurrent scleral buckling surgery. Cataract surgery was performed through a 2.4-mm self-sealing superior sclerocorneal tunnel. A continuous curvilinear capsulorhexis was performed, and the lens nucleus was removed, and the residual cortex was aspirated with an irrigation/aspiration tip. Next, a foldable acrylic intraocular lens was implanted into the bag. A trocar was then inserted at approximately 30° parallel to the limbus with the bevel-side up. After creating 3 ports, vitrectomy was performed using the Constellation^®^ system (Alcon Laboratories, Inc., Fort Worth, TX). After fluid-air exchange and subretinal fluid drainage from the causative retinal tear(s) or an iatrogenic hole, the causative retinal tear(s) or iatrogenic hole was photocoagulated. At the completion, 20% sulfur hexafluoride (SF_6_) was injected into the vitreous cavity. After the IOP was adjusted to a normal tension, the cannulae were withdrawn, and the sclera was pressed and massaged with an indenter to close the wound.

### Measurements of optical coherence tomographic images

A Spectralis SD-OCT instrument was used to obtain all SD-OCT images. We evaluated the horizontal cross-sectional images recorded at each visit after the successful retinal reattachment. As described in detail previously, [18] the thickness of the retinal layers was measured on the same selected central foveal scan throughout the follow-up period using the computer-based caliper measurement tool of the SD-OCT system. Briefly, the EZ-RPE thickness which is equivalent to the thickness of the outer segments (OS) of the photoreceptors was defined as the distance between the outer border of EZ and the inner border of RPE. [22] The integrity of the foveal ELM, EZ, and CIZ was evaluated for a 1-mm-diameter area of each image on a 4-point scale: 1, line not visible; 2, line disruption >200 μm; 3, line disruption <200 μm; and 4, continuous line. Identical measurements were performed on the fellow eyes as controls.

### Exclusion criteria

Eyes were excluded if; dense ocular media, e.g., severe cataract, vitreous hemorrhage, vitreous opacity were present, macular abnormalities, e.g., macular hole, vascular occlusive diseases, or diabetic retinopathy, were present, proliferative vitreoretinopathy (PVR) ≥grade C, [23] and clinically evident postoperative changes that were likely to interfere with accurate evaluation of retinal layers, e.g., recurrent RRD, epiretinal membrane, cystoid macular edema, or persistent subretinal fluid (SRF), were present. In addition, patients who had SRF at 2 weeks after the surgery were excluded because it was not possible to measure the retinal thickness especially the EZ-RPE thickness accurately.

### Statistical analyses

The values are presented as the means ± standard deviations. Independent *t* tests were used to compare the normally distributed data and the Chi-square test for the categorical data. The Pearson’s correlation coefficient test was used to determine the significance of the associations between each parameter and the BCVA. Multiple linear regression analyses were used to evaluate the association between the final BCVA and the perioperative independent variables.

## Results

### Demographics of patients and exclusion criteria

Between January 2012 to January 2015, 72 eyes of 72 patients with macula-off RRD underwent vitrectomy in our department for the repair of a RRD. Of these, 43 eyes were excluded for the following reasons; presence of proliferative vitreoretinopathy grade C or worse (n = 4), vitreous hemorrhage (n = 1), macular hole (n = 1), diabetic retinopathy (n = 1), postoperative development of dense cataract (n = 2), macular edema (n = 3), subretinal fluid (n = 6), significant epiretinal membrane (n = 1) at any time during the follow-up period with the presence of subretinal fluid for more than 2 weeks (n = 2), or an incapacity to attend regular follow-up visits (n = 22). As a result, 29 eyes with macula-off RRD were included in the final analyses. The demographics and surgical parameters of the patients are shown in Table 1.

**Table 1:** Patient clinical characteristics.

Characteristic	Macula-off
n (eyes)	29
Age (years)	61.3 ± 12.0
Males /females	18/11
LogMAR at the initial visit	1.29 ± 0.76
Axial length (mm)	25.2 ± 2.3
PPV/PPV+PEA+IOL	16/13

LogMAR, logarithm of the minimum angle of resolution; PPV, pars plana vitrectomy; PEA, phacoemulsification and aspiration; IOL, intraocular lens

### Changes of BCVA following vitrectomy in eyes with macula-off RRD

**Fig 1:**
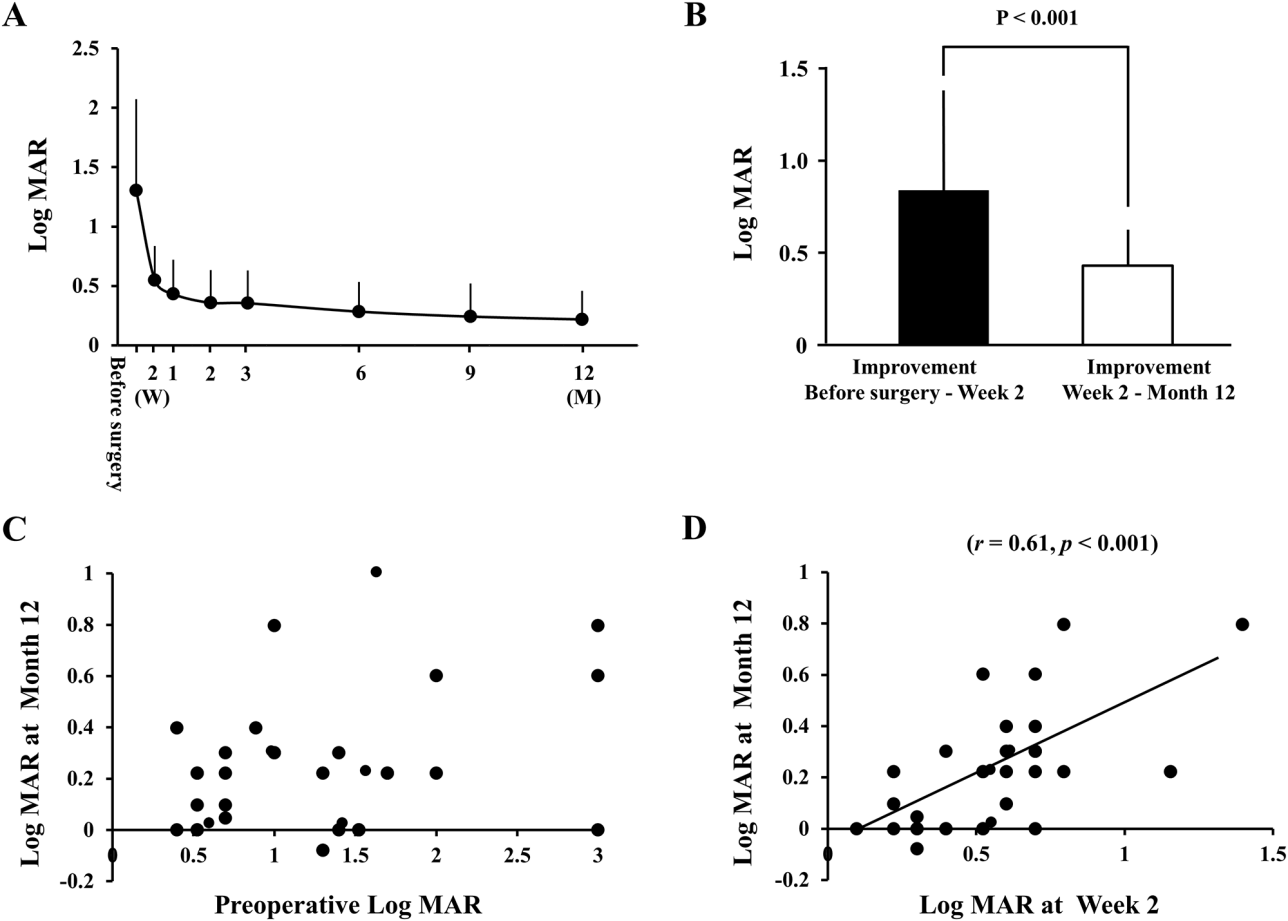
Changes in the best-corrected visual acuity (BCVA) in eyes with macula-off rhegmatogenous retinal detachment (RRD) following vitrectomy. The mean BCVA significantly improved with time (*P* <0.001); rapidly in the early postoperative period and more gradually in the later postoperative period (A). The improvement of the BCVA from that at preoperative to Week 2 was significantly greater than that from Week 2 to Month 12 (*P* < 0.001) (B). The final BCVA was not correlated with the preoperative BCVA (C), but was significantly correlated with that at Week 2 (*r* = 0.61, *P* <0.001) (D).

In eyes with macula-off RRD, the mean BCVA was 1.29 ± 0.76 logMAR units before the surgery, 0.55 ± 0.28 logMAR units at 2 weeks, 0.43 ± 0.28 logMAR units at 1 month, 0.36 ± 0.27 logMAR units at 2 months, 0.36 ± 0.27 logMAR units at 3 months, 0.29 ± 0.25 logMAR units at 6 months, 0.24 ± 0.28 logMAR units at 9 months, and 0.22 ± 0.24 logMAR units at 12 months following the surgery. The BCVA was significantly better than the preoperative BCVA at all times (*P* <0.001; Fig 1A). The mean BCVA in eyes affected by macula-off RRD at 12 months remained poorer than that of the fellow eyes (0.00 ± 0.13 logMAR units) at 12 months following the vitrectomy (*P* <0.001).

The improvement of the BCVA from the preoperative BCVA to Week 2 (-0.74 ± 0.69 logMAR units) was the largest improvement between consecutive observational periods, and it was significantly better than that from Week 2 to Month 12 (-0.31 ± 0.22 logMAR units; *P* <0.001; Fig 1B). The improvement of the BCVA became smaller with increasing time, and the improvement of BCVA from Week 2 to Month 1 was the second largest but the improvement was not significantly larger than that between Month 1 to Month 2.

### Changes of EZ-RPE thickness and the integrity of retinal microstructures of photoreceptors following surgery for macula-off RRD

**Fig 2:**
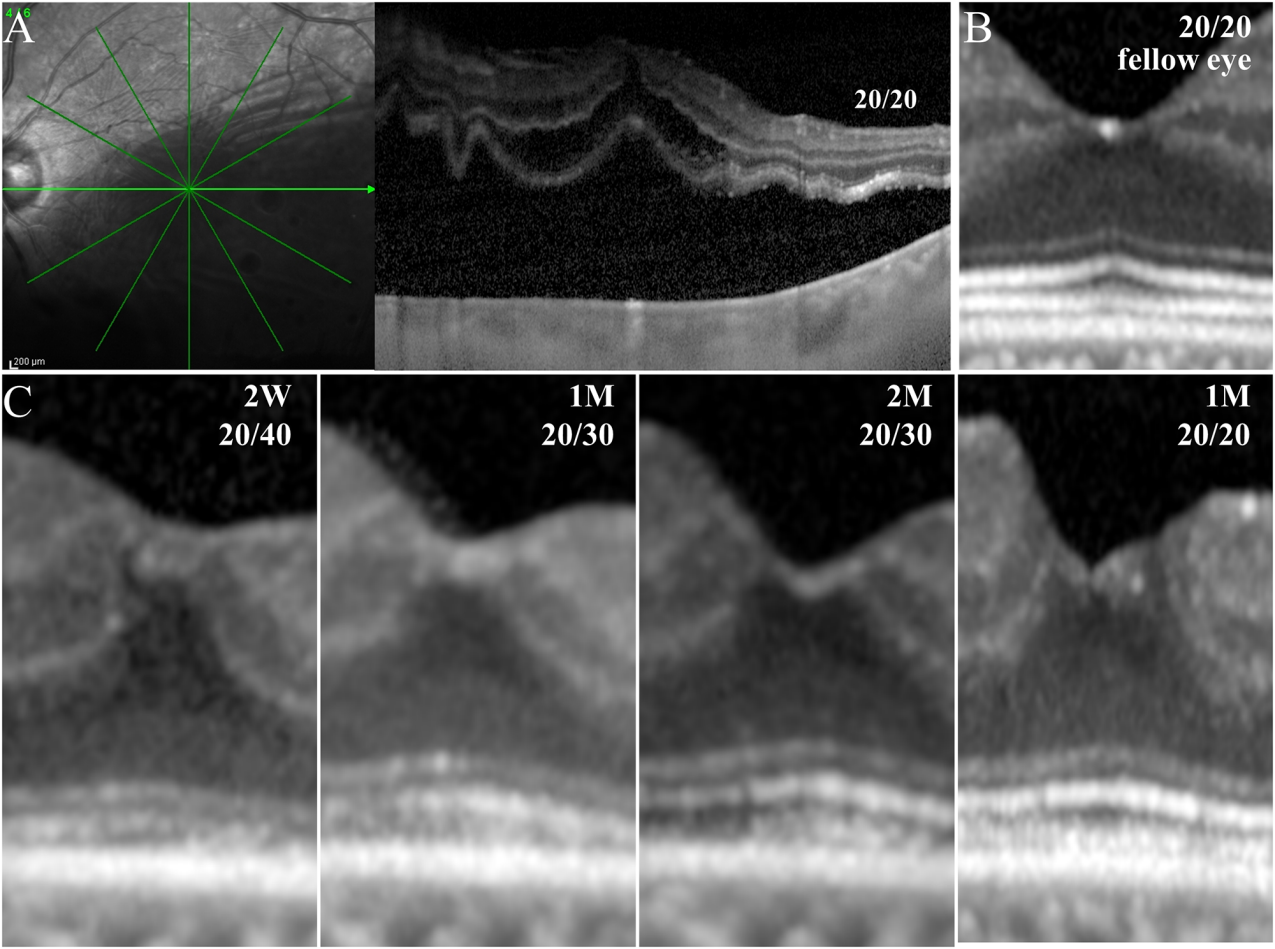
**Representative SD-OCT image of an eye with macula-off RRD before surgery (A). The thickness and reflectivity of the outer retinal bands were normal in the unaffected fellow eye (B). The ELM, EZ, and CIZ appeared fragmented and thin at 2 weeks after surgery (C).** A progressive increase in the reflectivity of the outer bands can be seen with time. The length and reflectivity of the outer bands became similar to that of the fellow eye at 12 months postoperatively. The thickness of the outer bands remained thinner than that of the corresponding bands in the fellow eye (C).

The restoration of the retinal microstructures after surgery is shown in Fig 2. In eyes affected by macula-off RRD, the mean EZ-RPE thickness was 6.6 ± 7.8 μm at 2 weeks, 18.5 ± 13.1 μm at 1 month, 26.6 ± 14.7 μm at 2 months, 30.2 ± 12.8 μm at 3 months, 33.8 ± 10.9 μm at 6 months, 34.5 ± 11.4 μm at 9 months, and 36.2 ± 10.6 μm at 12 months following the surgery.

**Fig 3:**
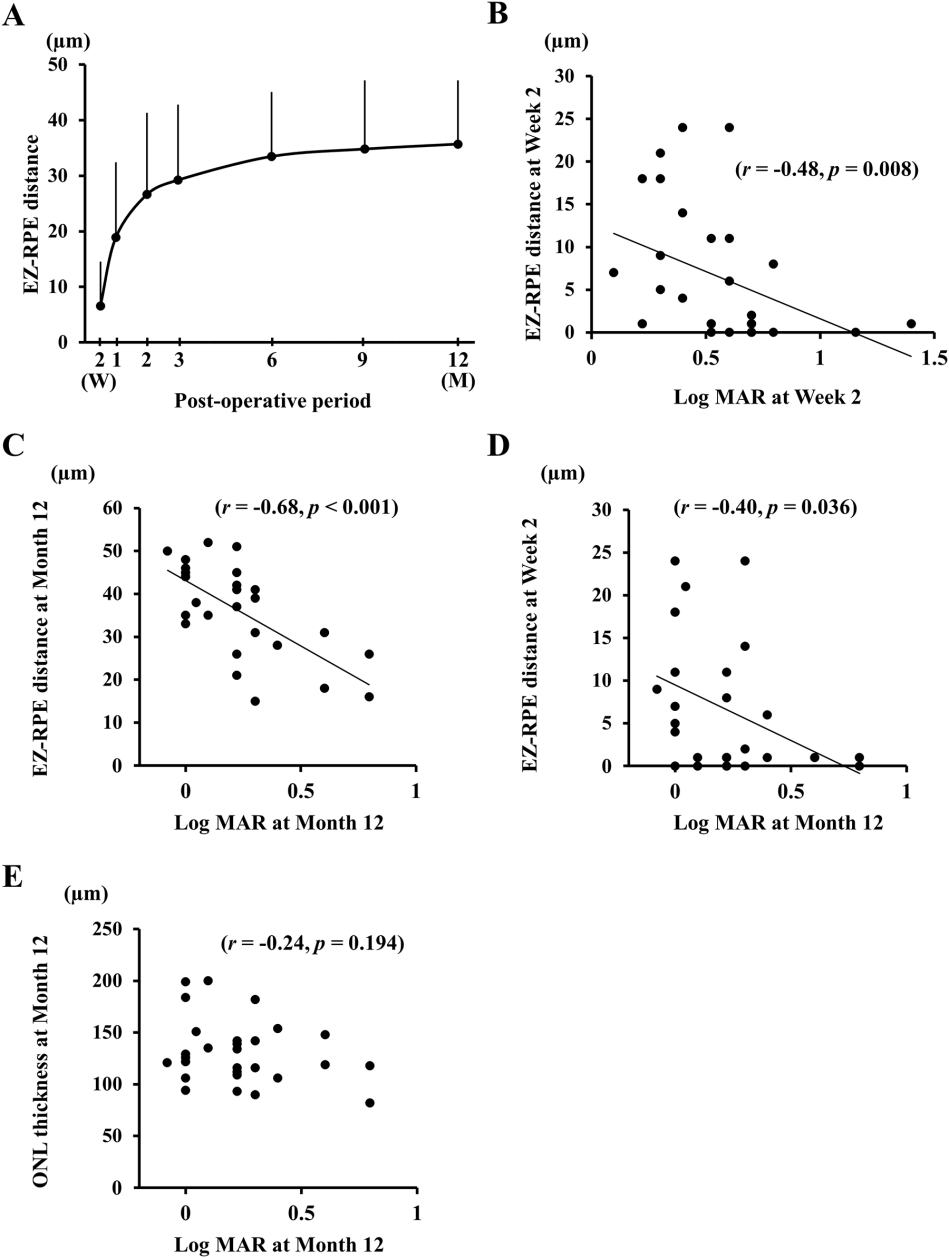
Changes in mean EZ–RPE thickness following surgery, and the correlation between the thickness and the BCVA is shown for eyes with macula-off RRD. The mean EZ-RPE thickness increases significantly over time (*P* < 0.001), and it is especially rapid in the early postoperative period (A). The EZ-RPE thicknesses at Week 2 and Month 12 were significantly correlated with the BCVA at Week 2 (*r* = -0.48, *P* = 0.008) (B) and at Month 12 (*r* = -0.68, *P* < 0.001) (C), respectively. The EZ-RPE thickness at Week 2 was significantly correlated with the BCVA at Month 12 (*r* = -0.40, *P* = 0.036) (D). The foveal outer nuclear layer (ONL) thickness at Month 12 was not correlated with the BCVA at Month 12 (E).

The increased thickness with time was significant (*P* < 0.001; Fig 3A). The mean EZ-RPE at Week 2 was significantly thinner than that at Month 1 (*P* < 0.001), and the increase in the thickness from Week 2 to Month 1 following surgery (12.0 ± 11.3 μm) was the largest between adjacent observational periods (Fig 3).

The mean scores of the integrity of the ELM, EZ, and CIZ at Week 2 were 3.4 ± 0.8, 2.4 ± 0.9, and 1.1 ± 0.2, respectively.

### Correlation between the perioperative parameters and the BCVA at Month 12

The BCVA at Month 12 was not significantly correlated with the preoperative BCVA but was significantly correlated with that at Week 2 (*r* = 0.61, *P* <0.001), Month 1 (*r* = 0.75, *P* <0.001), Month 2 (*r* = 0.62, *P* <0.001), Month 3 (*r* = 0.81, *P* <0.001), Month 6 (r = 0.92, *P* <0.001), and Month 9 (*r* = 0.91, *P* <0.001; Fig 1). The BCVA at Week 2 was significantly correlated with the EZ-RPE thickness at Week 2 (*r* = -0.48, *P* = 0.008). In addition, the BCVA at Month 12 was significantly correlated with the EZ-RPE thickness at Month 12 (*r* = -0.68, *P* < 0.001). None of the preoperative parameters were significantly correlated with the final BCVA. The final BCVA was correlated with the EZ-RPE thickness at Week 2 (*r* = -0.40, *P* = 0.036) and the integrity of the ELM (*r* = -0.61, *P* = 0.003) and the EZ (*r* = -0.62, *P* = 0.001) at Week 2.

Multiple stepwise regression analysis of the final BCVA (Table 2) showed that the BCVA at Week 2 (*P* = 0.017) and the integrity of the EZ at Week 2 were independent and significant factors associated with the final BCVA (*P* = 0.006).

**Table 2:** Results of multiple stepwise regression analysis for independence of factors contributing to final BCVA.

Variable			
Dependent	Independent	*β*	*p*-value
Final BCVA	EZ at Week 2	-0.523	0.006
	BCVA at Week 2	0.426	0.017
	BCVA before surgery	0.423	0.063
	Age	-0.152	0.522
	Integrity of CIZ at Week 2	0.128	0.591
	Integrity of ELM at Week 2	-0.108	0.651
	Gender	-0.023	0.923
	Axial length	0.008	0.974
	EZ-RPE thickness at Week 2	-0.006	0.979

BCVA, best-corrected visual acuity; EZ, ellipsoid zone; RPE, retinal pigment epithelium; CIZ, cone inter digitation zone; ELM, external limiting membrane

## Discussion

Our results showed that the largest improvement of the BCVA was between the preoperative BCVA and that at Week 2 of any adjacent observation periods, and it was even greater than that from Week 2 to Month 12. The BCVA at Week 2 was significantly and positively correlated with that at Month 12, while the preoperative BCVA was not correlated with the BCVA at Month 12. The EZ-RPE thickness and the integrity of the ELM and the EZ at Week 2 were significantly correlated with the BCVA at Month 12. Multiple regression analysis showed that the BCVA and the integrity of EZ at Week 2 were independent predictors of the final BCVA.

The BCVA at Week 2 was already significantly correlated with the final vision although the correlation between the preoperative and final vision was not significant. In addition, the improvement of visual acuity before surgery to Week 2 was greater than that from Week 2 to Month 12. Week 2 is very early in the recovery period, but the retina was attached successfully and the SRF was not present at the fovea at this time in all of the eyes. These results indicate that the detachment of the fovea was the cause of the reduced visual acuity.

Although many factors have been found to influence visual recovery in eyes with a macula-off RRD, the most important predictor for visual recovery after macula-off RRD surgery has been reported to be the preoperative vision.[7, 24–26] However, it was not significantly correlated with the final vision in the present study. We do not know why our results are different from the earlier results, but perhaps the number of patients we studied was too few to evaluate this factor. However, it is more likely that the other preoperative factors, e.g. patients’ age [4] and duration of detachment, [8] [9] and height of macular detachment, [10] [11] were more important factors that contributed to the final visual acuity. Accordingly, the status of the many factors contributing to the preoperative vision probably was the reason why no significant correlation was found between the preoperative and the final BCVA.

Taken together, our results showed that the preoperative BCVA is affected by the several factors mentioned, and some of these factors, e.g. height of macular detachment are resolved by the re-attachment of retina. This would then cause a significant improvement of the BCVA within 2 weeks following surgery and positive correlation in BCVA between Week 2 and Month 12.

Recent improvements in the resolution of OCT instruments have made it possible to obtain more precise and accurate evaluations of the retinal microstructures not only the integrity of each structure but also a quantitative analysis on the length across the photoreceptor layer.[18, 20, 21] We evaluated the microstructures of the photoreceptors and also measured the EZ-RPE thickness, i.e., the OS length. The mean EZ-RPE thickness was 6.6 μm at Week 2 and increased to 36.5 μm at Month 12. Similar to the improvement of vision, the EZ-RPE thickness increased rapidly in the early postoperative period and more slowly in the later postoperative period.

There have been several reports describing a thinning of the retinal layers following RRD. Experimental studies have demonstrated a loss of the OS of the photoreceptors due to the separation of the photoreceptors from the RPE, thereby disrupting normal OS renewal and leading to OS shortening and eventual degeneration. [27–29] A variety of changes in several cell types throughout the retina can be caused by detachment of the neural retina from the RPE. The thickness of EZ-RPE at Week 2 was correlated with the BCVA at Week 2. Dell’Omo et al. and Terauchi et al. [21] reported that the OS thickness at Month 1 was significantly correlated with the BCVA at Month 1. Thus, it was not surprising to find a significant correlation between the EZ-RPE thickness and the BCVA even in the very early postoperative period of Week 2. Interestingly, the thickness of EZ-RPE at Week 2 was correlated with the BCVA at Month 12. It has been reported that the increase in the OS length is correlated with the improvement of BCVA.[18] Taken together, eyes with better vision at Week 2 would have longer OSs from the very early postoperative period, and the elongation led to the improvement of the BCVA with time. This would then account for the significant correlation between the thickness of EZ-RPE at Week 2 and the final vision.

Many studies using SD-OCT have demonstrated that integrity of retinal microstructures is significantly correlated with the BCVA following retinal reattachment.[12–18] It has been reported that the integrity of CIZ is an important factor in obtaining better vision throughout the postoperative period. [18] However, the EZ-RPE is relatively thin at Week 2, and it is difficult to observe the CIZ which is present between the EZ and the RPE in most of eyes using the images recorded with a SD-OCT. On the other hand, the ELM is visible in most of eyes even at Week 2, and the multiple regression analyses showed that the integrity of EZ at a very early postoperative period would be a good predictor of the final BCVA.

Surgeons still cannot accurtely predict the postoperative BCVAs in eyes with macula-off RRD in the perioperative period. Our results suggest that predicting the final BCVA is difficult before surgery because of the many factors that can affect the preoperative vision, but the findings of the BCVA and the integrity of EZ at Week 2 can be good candidates to predict the final BCVA.

There are limitations to this study. First, this was a retrospective study on a relatively small sample which would lower the statistical power of the findings. Second, we did not evaluate the preoperative microstructure of retinal layers and the thickness of retinal layers, because it is difficult to observe these structures accurately in the preoperative SD-OCT images. Therefore, it is unclear whether eyes with longer OSs at Week 2 already had longer OSs preoperatively or had short OSs but they quickly elongated by 2 weeks following surgery. Third, the retinal layer thicknesses were measured manually because automated calculation of retinal layer thicknesses is difficult in eyes which the retinal layers are fragmented and not clearly distinguishable. Fourth, we performed vitrectomy combined with cataract surgery for some patients, but eyes with severe cataract formation were excluded. Accordingly, the vision improvement by cataract surgery should be small in this study. Further prospective studies on a larger number of cases with automated calculation of retinal thicknesses will be necessary to be validated that a good BCVA and the integrity of EZ at week 2 can be suggested as a predictor of long-term visual outcome in eyes following RRD surgery.

In conclusion, the BCVA has improved significantly by 2 weeks following vitrectomy. A good BCVA and the integrity of EZ at week 2 can be good candidates for predicting the final BCVA following vitrectomy in eyes with a macula-off RRD.

## Supporting information

S1 File Dataset. (XLSX)

## References

[1] D'Amico DJ. Clinical practice. Primary retinal detachment. N Engl J Med. 2008;359(22):2346–54. Epub 2008/11/29. doi: 10.1056/NEJMcp0804591 . 19038880 10.1056/NEJMcp0804591

[2] Goto T, Nakagomi T, Iijima H. A comparison of the anatomic successes of primary vitrectomy for rhegmatogenous retinal detachment with superior and inferior breaks. Acta Ophthalmol. 2013;91(6):552–6. doi: 10.1111/j.1755-3768.2012.02455.x . 22691313 10.1111/j.1755-3768.2012.02455.x

[3] Campo RV, Sipperley JO, Sneed SR, Park DW, Dugel PU, Jacobsen J, et al Pars plana vitrectomy without scleral buckle for pseudophakic retinal detachments. Ophthalmology. 1999;106(9):1811–5; discussion 6. Epub 1999/09/15. doi: 10.1016/S0161-6420(99)90353-6 . 10485555 10.1016/S0161-6420(99)90353-6

[4] Tan HS, Oberstein SY, Mura M, Bijl HM. Air versus gas tamponade in retinal detachment surgery. Br J Ophthalmol. 2013;97(1):80–2. Epub 2012/10/30. doi: 10.1136/bjophthalmol-2012-302140 . 23104901 10.1136/bjophthalmol-2012-302140

[5] Sabates NR, Sabates FN, Sabates R, Lee KY, Ziemianski MC. Macular changes after retinal detachment surgery. Am J Ophthalmol. 1989;108(1):22–9. Epub 1989/07/15. . 2750831 10.1016/s0002-9394(14)73255-6

[6] Mervin K, Valter K, Maslim J, Lewis G, Fisher S, Stone J. Limiting photoreceptor death and deconstruction during experimental retinal detachment: the value of oxygen supplementation. Am J Ophthalmol. 1999;128(2):155–64. Epub 1999/08/24. . 10458170 10.1016/s0002-9394(99)00104-x

[7] Tani P, Robertson DM, Langworthy A. Prognosis for central vision and anatomic reattachment in rhegmatogenous retinal detachment with macula detached. Am J Ophthalmol. 1981;92(5):611–20. Epub 1981/11/01. . 7304687 10.1016/s0002-9394(14)74651-3

[8] Diederen RM, La Heij EC, Kessels AG, Goezinne F, Liem AT, Hendrikse F. Scleral buckling surgery after macula-off retinal detachment: worse visual outcome after more than 6 days. Ophthalmology. 2007;114(4):705–9. Epub 2006/12/30. doi: 10.1016/j.ophtha.2006.09.004 . 17194479 10.1016/j.ophtha.2006.09.004

[9] Hassan TS, Sarrafizadeh R, Ruby AJ, Garretson BR, Kuczynski B, Williams GA. The effect of duration of macular detachment on results after the scleral buckle repair of primary, macula-off retinal detachments. Ophthalmology. 2002;109(1):146–52. Epub 2002/01/05. . 11772595 10.1016/s0161-6420(01)00886-7

[10] Ross W, Lavina A, Russell M, Maberley D. The correlation between height of macular detachment and visual outcome in macula-off retinal detachments of < or = 7 days' duration. Ophthalmology. 2005;112(7):1213–7. Epub 2005/06/01. doi: 10.1016/j.ophtha.2005.01.040 . 15921745 10.1016/j.ophtha.2005.01.040

[11] Mowatt L, Tarin S, Nair RG, Menon J, Price NJ. Correlation of visual recovery with macular height in macula-off retinal detachments. Eye (Lond). 2010;24(2):323–7. Epub 2009/04/25. doi: 10.1038/eye.2009.74 . 19390562 10.1038/eye.2009.74

[12] Gharbiya M, Grandinetti F, Scavella V, Cecere M, Esposito M, Segnalini A, et al Correlation between spectral-domain optical coherence tomography findings and visual outcome after primary rhegmatogenous retinal detachment repair. Retina. 2012;32(1):43–53. Epub 2011/07/23. doi: 10.1097/IAE.0b013e3182180114 . 21778929 10.1097/IAE.0b013e3182180114

[13] Smith AJ, Telander DG, Zawadzki RJ, Choi SS, Morse LS, Werner JS, et al High-resolution Fourier-domain optical coherence tomography and microperimetric findings after macula-off retinal detachment repair. Ophthalmology. 2008;115(11):1923–9. Epub 2008/08/02. doi: 10.1016/j.ophtha.2008.05.025 . 18672289 10.1016/j.ophtha.2008.05.025 PMC2735404

[14] Schocket LS, Witkin AJ, Fujimoto JG, Ko TH, Schuman JS, Rogers AH, et al Ultrahigh-resolution optical coherence tomography in patients with decreased visual acuity after retinal detachment repair. Ophthalmology. 2006;113(4):666–72. Epub 2006/04/04. doi: 10.1016/j.ophtha.2006.01.003 . 16581427 10.1016/j.ophtha.2006.01.003 PMC1940045

[15] Wakabayashi T, Oshima Y, Fujimoto H, Murakami Y, Sakaguchi H, Kusaka S, et al Foveal microstructure and visual acuity after retinal detachment repair: imaging analysis by Fourier-domain optical coherence tomography. Ophthalmology. 2009;116(3):519–28. Epub 2009/01/17. doi: 10.1016/j.ophtha.2008.10.001 . 19147231 10.1016/j.ophtha.2008.10.001

[16] Itoh Y, Inoue M, Rii T, Hiraoka T, Hirakata A. Significant correlation between visual acuity and recovery of foveal cone microstructures after macular hole surgery. Am J Ophthalmol. 2012;153(1):111–9.e1. Epub 2011/09/02. doi: 10.1016/j.ajo.2011.05.039 . 21880295 10.1016/j.ajo.2011.05.039

[17] Srinivasan VJ, Monson BK, Wojtkowski M, Bilonick RA, Gorczynska I, Chen R, et al Characterization of outer retinal morphology with high-speed, ultrahigh-resolution optical coherence tomography. Invest Ophthalmol Vis Sci. 2008;49(4):1571–9. Epub 2008/04/04. doi: 10.1167/iovs.07-0838 . 18385077 10.1167/iovs.07-0838 PMC2846094

[18] Kobayashi M, Iwase T, Yamamoto K, Ra E, Murotani K, Matsui S, et al Association Between Photoreceptor Regeneration and Visual Acuity Following Surgery for Rhegmatogenous Retinal Detachment. Invest Ophthalmol Vis Sci. 2016;57(3):889–98. Epub 2016/03/05. doi: 10.1167/iovs.15-18403 . 26943151 10.1167/iovs.15-18403

[19] Hasegawa T, Ueda T, Okamoto M, Ogata N. Relationship between presence of foveal bulge in optical coherence tomographic images and visual acuity after rhegmatogenous retinal detachment repair. Retina. 2014;34(9):1848–53. Epub 2014/04/20. doi: 10.1097/IAE.0000000000000160 . 24743639 10.1097/IAE.0000000000000160

[20] dell'Omo R, Viggiano D, Giorgio D, Filippelli M, Di Iorio R, Calo R, et al Restoration of foveal thickness and architecture after macula-off retinal detachment repair. Invest Ophthalmol Vis Sci. 2015;56(2):1040–50. Epub 2015/01/24. doi: 10.1167/iovs.14-15633 . 25613940 10.1167/iovs.14-15633

[21] Terauchi G, Shinoda K, Matsumoto CS, Watanabe E, Matsumoto H, Mizota A. Recovery of photoreceptor inner and outer segment layer thickness after reattachment of rhegmatogenous retinal detachment. Br J Ophthalmol. 2015 Epub 2015/04/05. doi: 10.1136/bjophthalmol-2014-306252 . 25841234 10.1136/bjophthalmol-2014-306252

[22] Staurenghi G, Sadda S, Chakravarthy U, Spaide RF. Proposed lexicon for anatomic landmarks in normal posterior segment spectral-domain optical coherence tomography: the IN*OCT consensus. Ophthalmology. 2014;121(8):1572–8. Epub 2014/04/24. doi: 10.1016/j.ophtha.2014.02.023 . 24755005 10.1016/j.ophtha.2014.02.023

[23] Machemer R, Aaberg TM, Freeman HM, Irvine AR, Lean JS, Michels RM. An updated classification of retinal detachment with proliferative vitreoretinopathy. Am J Ophthalmol. 1991;112(2):159–65. . 1867299 10.1016/s0002-9394(14)76695-4

[24] Sharma T, Challa JK, Ravishankar KV, Murugesan R. Scleral buckling for retinal detachment. Predictors for anatomic failure. Retina. 1994;14(4):338–43. Epub 1994/01/01. . 7817027 10.1097/00006982-199414040-00008

[25] Oshima Y, Yamanishi S, Sawa M, Motokura M, Harino S, Emi K. Two-year follow-up study comparing primary vitrectomy with scleral buckling for macula-off rhegmatogenous retinal detachment. Jpn J Ophthalmol. 2000;44(5):538–49. Epub 2000/10/18. . 11033134 10.1016/s0021-5155(00)00205-7

[26] Grizzard WS, Hilton GF, Hammer ME, Taren D. A multivariate analysis of anatomic success of retinal detachments treated with scleral buckling. Graefes Arch Clin Exp Ophthalmol. 1994;232(1):1–7. Epub 1994/01/01. . 8119596 10.1007/BF00176431

[27] Lewis GP, Charteris DG, Sethi CS, Leitner WP, Linberg KA, Fisher SK. The ability of rapid retinal reattachment to stop or reverse the cellular and molecular events initiated by detachment. Invest Ophthalmol Vis Sci. 2002;43(7):2412–20. Epub 2002/07/02. . 12091445

[28] Sakai T, Calderone JB, Lewis GP, Linberg KA, Fisher SK, Jacobs GH. Cone photoreceptor recovery after experimental detachment and reattachment: an immunocytochemical, morphological, and electrophysiological study. Invest Ophthalmol Vis Sci. 2003;44(1):416–25. Epub 2002/12/31. . 12506104 10.1167/iovs.02-0633

[29] Jackson TL, Hillenkamp J, Williamson TH, Clarke KW, Almubarak AI, Marshall J. An experimental model of rhegmatogenous retinal detachment: surgical results and glial cell response. Invest Ophthalmol Vis Sci. 2003;44(9):4026–34. Epub 2003/08/27. . 12939325 10.1167/iovs.02-1264

